# Diploids derived from polyploids: genetic characteristics of four novel interspecific *Sorghum* populations

**DOI:** 10.1093/g3journal/jkag165

**Published:** 2026-07-07

**Authors:** Wenqian Kong, Pheonah Nabukalu, Stan Cox, Jaxk Reeves, Andrew H Paterson

**Affiliations:** Plant Genome Mapping Laboratory, Institute of Plant Breeding, Genetics & Genomics (IPBGG), University of Georgia, Athens, GA 30602, United States; Department of Statistics, University of Georgia, Athens, GA 30602, United States; The Land Institute, Salina, KS 67401, United States; The Land Institute, Salina, KS 67401, United States; Department of Statistics, University of Georgia, Athens, GA 30602, United States; Plant Genome Mapping Laboratory, Institute of Plant Breeding, Genetics & Genomics (IPBGG), University of Georgia, Athens, GA 30602, United States; Department of Crop, Soil and Environmental Sciences, Auburn University, Auburn, AL 36849, United States

**Keywords:** polyploidy, diploidization, *Sorghum*, introgression, segregation distortion, QTL mapping

## Abstract

Polyploidy has repeatedly shaped grass evolution, yet direct observations of how polyploid-derived chromosomes behave when returned to diploidy remain rare. Interspecific crosses between diploid *Sorghum bicolor* and tetraploid hybrids derived from *Sorghum halepense* generate mixed-ploidy progeny, providing an opportunity to examine chromosome transmission during the early stages of diploidization. Using genome-wide SNP markers, we characterized chromosomal inheritance patterns in 2 diploid and 2 tetraploid families derived from these crosses. Genotype-dosage profiles alone distinguished diploids from tetraploids with complete accuracy, reflecting strong ploidy-dependent differences in dosage-class distributions. Although diploid progeny retained much of the halepense-derived genomic background, several genomic intervals exhibited extended, nonrandom runs of *S. bicolor* homozygosity that remained polymorphic in corresponding tetraploid populations. These patterns, together with recurrent segregation distortion across independent families, suggest that the transition from tetraploidy to diploidy can expose allelic combinations that differ in transmission or viability. Analyses of flowering time further indicated that diploid and tetraploid derivatives possess distinct genomic architectures, with major association peaks occurring in different chromosomal regions across ploidy levels. Collectively, these results indicate that early diploidization involves nonrandom retention and loss of parental haplotypes shaped by both selective and structural constraints. The diploid extractions characterized here provide a rare empirical system for investigating the early stages of diploidization and a practical framework for studying and eventually mobilizing polyploid-derived variation for sorghum germplasm development. However, broader integration into elite breeding programs will require additional evaluation of cross-fertility, meiotic behavior, and chromosomal stability across diverse breeding backgrounds.

## Teaser

Polyploid genomes have played a major role in crop evolution, yet little is known about chromosome behavior when polyploid-derived genomes return to diploidy. This study examined four interspecific sorghum populations derived from crosses between diploid Sorghum bicolor and tetraploid Sorghum halepense-derived hybrids. Using genome-wide SNP markers, Kong et al. compared chromosomal inheritance, genomic composition, and flowering-time architecture across diploid and tetraploid derivatives. The authors identified non-random retention and loss of parental genomic regions and demonstrated that dosage-based genotypes accurately distinguish ploidy classes. These findings provide insight into early diploidization and establish a framework for studying and mobilizing polyploid-derived variation for sorghum improvement.

## Introduction

Polyploidy has played a central role in angiosperm evolution, repeatedly generating duplicated chromosome sets that introduce new opportunities-–and constraints-–for genome function. Newly formed polyploids often exhibit instability as duplicated loci, homoeologous chromosomes, and dosage relationships interact in ways not encountered in diploids. Polyploid lineages that persist must therefore stabilize the behavior of multiple chromosome complements within a shared nucleus. This stabilization typically involves coordinated gene loss, chromosomal restructuring, and reductions in chromosome number that collectively drive the return to a more diploid-like state. Such diploidization processes can influence the ecological range, competitive ability, and evolutionary potential of polyploids, helping explain their disproportionate representation among invasive plant taxa (Pandit et al. 2006).

Polyploids also frequently differ from their diploid relatives in morphology, physiology, and genomic composition (Parisod et al. 2010). A well-studied example within the grasses is *Sorghum halepense* (johnsongrass), an allotetraploid species (2n=4x=40) formed through hybridization between diploid *Sorghum bicolor* and *Sorghum propinquum*. The species combines vigorous rhizomatous growth, recurrent flowering, and broad ecological adaptability, and it hybridizes with cultivated sorghum at documented outcrossing rates generally below 10% (Arriola and Ellstrand 1996, 1997; Morrell et al. 2005). These characteristics contribute to persistent crop–weed gene flow and illustrate how polyploid genomes can integrate genetic material from divergent parental species into genomic combinations that enhance both adaptability and invasiveness.

Despite its reputation as a weed, *S. halepense* harbors alleles of considerable value for sorghum improvement. Traits such as perenniality, rhizome development, and abiotic-stress tolerance have motivated efforts to introgress halepense-derived segments into *S. bicolor*. Early work at The Land Institute showed that interspecific tetraploid populations produced via unreduced-gamete formation capture extensive phenotypic and genomic variation relevant to perennial grain development (Piper and Kulakow 1994; Kong 2017; Cox et al. 2018b). Yet tetraploid genomes introduce analytical challenges: polysomic inheritance obscures segregation patterns, recombination among homoeologous chromosomes complicates map construction, and introgressed segments may be difficult to trace or transfer into diploid breeding lines.

A striking observation from these breeding efforts was that certain tetraploid intermediates occasionally produce viable diploid offspring when crossed to diploid *S. bicolor* (Cox et al. 2018a). These “diploid extractions” effectively return polyploid-derived chromosomal segments to a diploid genomic context. Such events create an unusually tractable system for studying the early stages of diploidization by allowing direct comparison of homologous chromosomal regions maintained in both diploid and tetraploid backgrounds. Systems that capture this transition from polyploid to diploid inheritance with shared parental genomes are exceedingly rare in plants.

**Fig. 1.**
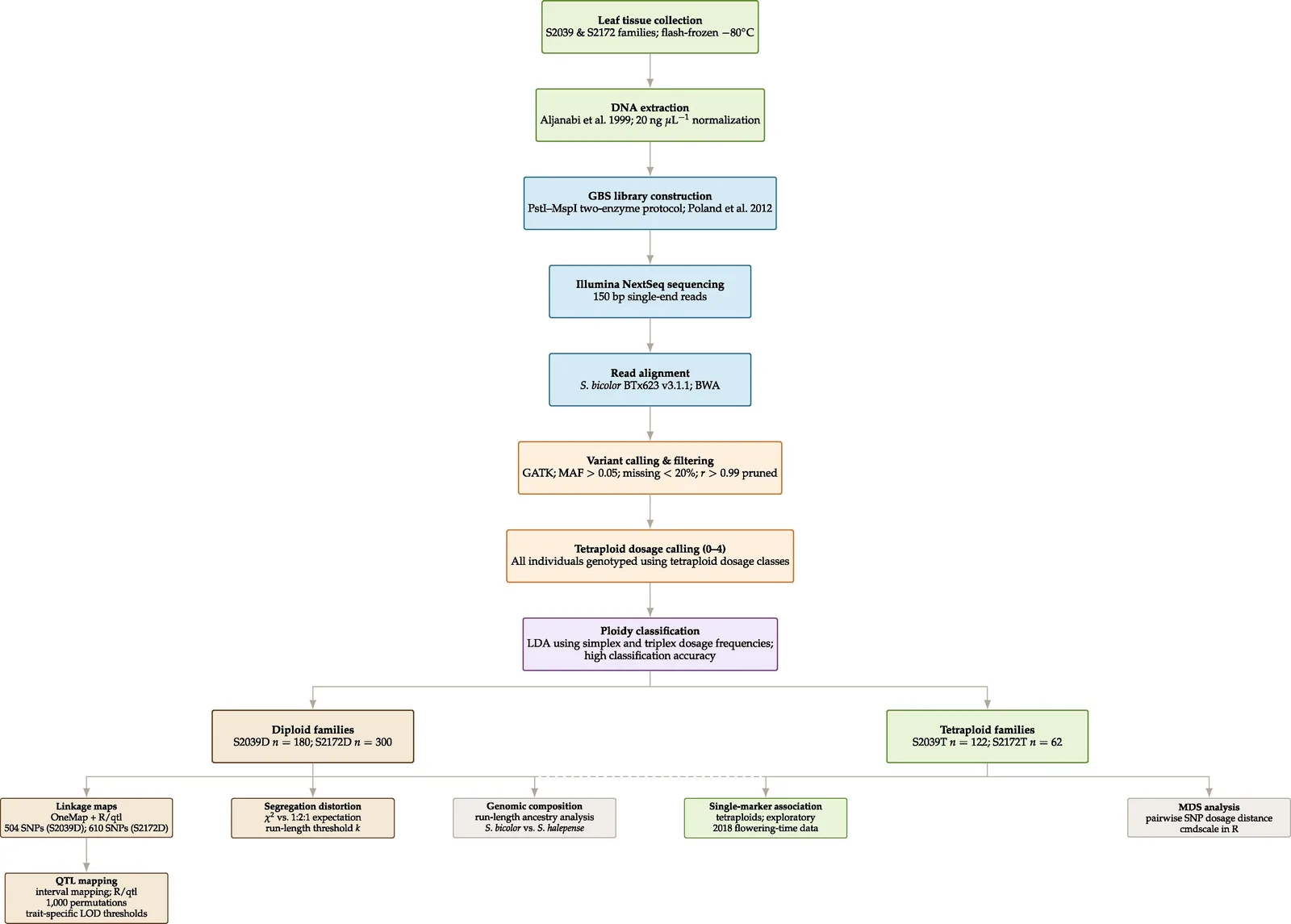
Overview of the analytical workflow used for characterization of the S2039 and S2172 diploid and tetraploid sorghum populations. Leaf tissue was processed using a genotyping-by-sequencing (GBS) pipeline followed by SNP filtering, tetraploid dosage calling (dosage classes 0–4), and ploidy classification by linear discriminant analysis (LDA) using simplex and triplex dosage frequencies. Diploid families were analyzed using linkage mapping (OneMap; R/qtl), segregation distortion ($\chi^{2}$ vs. 1:2:1 expectation), run-length ancestry analysis, and QTL mapping by interval mapping with 1,000-permutation, trait-specific LOD thresholds. Tetraploid families were used for exploratory single-marker association analyses of 2018 flowering time (Bonferroni α=0.05). Dashed arrows indicate analyses performed jointly across both ploidy classes.

In this study, we characterize 2 diploid and 2 tetraploid interspecific families derived from crosses between diploid *S. bicolor* and *S. halepense*-derived tetraploids. Using genome-wide SNP markers, we (i) quantify patterns of parental haplotype transmission, (ii) identify genomic regions exhibiting biased introgression or segregation distortion, (iii) compare the retention and organization of *S. halepense*- versus *S. bicolor*-derived segments across ploidy levels, and (iv) resolve the genetic architecture of flowering time (FT) in both diploid and tetraploid derivatives. By jointly analyzing diploid extractions and their tetraploid progenitors, we gain insight into chromosome behavior as polyploid-derived alleles are returned to diploidy, advancing understanding of early diploidization processes while identifying polyploid-origin alleles with potential value for sorghum improvement. An overview of the complete analytical workflow is presented in Fig. 1.

## Materials and methods

### Population development

**Fig. 2.**
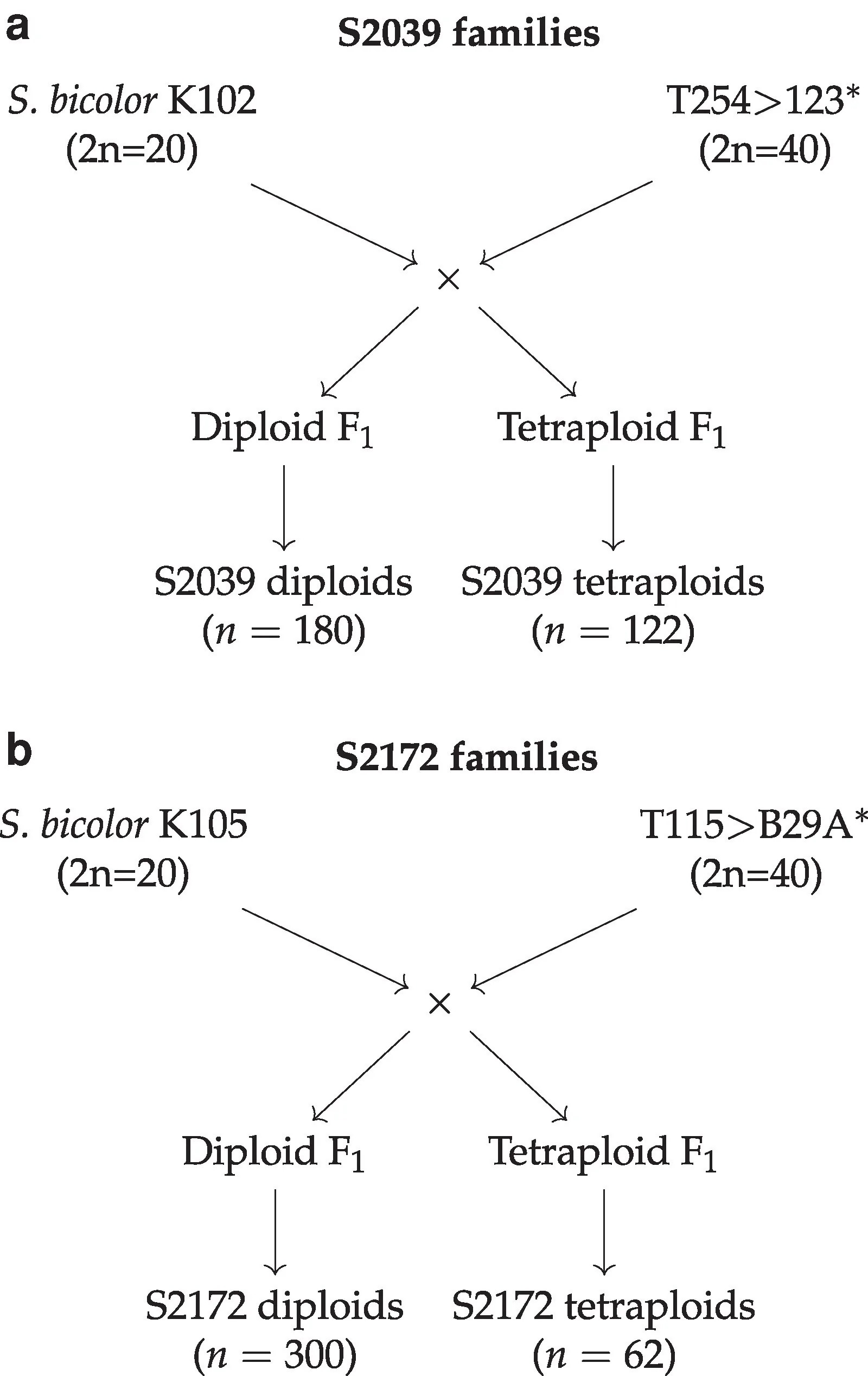
Parental and progeny relationships for the S2039 and S2172 diploid and tetraploid families. Diploid *S. bicolor* lines K102 and K105 were crossed to tetraploid intermediates T254>123 and T115>B29A, respectively. Mixed-ploidy $F_{1}$ progeny segregated into diploid and tetraploid classes, which were advanced to generate the S2039 and S2172 families; *n* reflects individuals retained after genotyping quality control. ^*^Tetraploid intermediates were produced by recurrent backcrossing of *S. halepense* to cultivated *S. bicolor*. a) S2039 families and b) S2172 families.

We analyzed 2 related interspecific families, each comprising paired diploid and tetraploid populations derived from crosses between cultivated diploid *S. bicolor* and *S. halepense*-derived tetraploids. The relationships among parental lines and the segregation of mixed-ploidy $F_{1}$ progeny into $F_{2}$ diploid and tetraploid families are schematized in Fig. 2.

The S2039 families trace to a cross between diploid *S. bicolor* K102 (2n=20) and tetraploid T254>123 (hereafter T254; 2n=40), an intermediate developed by recurrently backcrossing *S. halepense* to *S. bicolor*. Segregation of ploidy in the $F_{1}$ generation yielded a diploid $F_{2}$ family (S2039D; N=180) and a tetraploid $F_{2}$ family (S2039T; N=122).

Similarly, the S2172 families were obtained by crossing diploid *S. bicolor* K105 with tetraploid T115>B29A (hereafter T115), another intermediate generated by repeated introgression of *S. halepense* into a cultivated sorghum background. The resulting $F_{2}$ progeny segregated into 300 diploid individuals (S2172D) and 62 tetraploid individuals (S2172T). Details of tetraploid intermediate development and recovery of diploid progeny from interploid crosses are described in Cox et al. (2018a).

### Genotyping-by-sequencing

Leaf samples from all individuals were flash-frozen at $-80^{\circ }$C and lyophilized for 48 h prior to DNA extraction. Genomic DNA was isolated using the rapid, polysaccharide- and polyphenol-free protocol of Aljanabi et al. (1999). GBS libraries were constructed following the two-enzyme PstI–MspI protocol of Poland et al. (2012). DNA concentrations were normalized to $20\;\mathrm{ng}\;\mu L^{-1}$ before library preparation. Amplified libraries were size-selected to 250–300 bp using the QIAquick Gel Extraction Kit (QIAGEN).

Libraries were sequenced on an Illumina NextSeq platform (RRID:SCR_016400) with 150 bp single-end reads. Sequence reads were aligned to the *S. bicolor* BTx623 v3.1.1 reference genome (McCormick et al. 2018) using the Burrows–Wheeler Aligner (RRID:SCR_010910) (Li and Durbin 2009). Variant discovery and initial quality filtering were performed with the Genome Analysis Toolkit (GATK; RRID:SCR_001876) (McKenna et al. 2010).

### Phenotyping for FT

The four populations (S2039D, S2039T, S2172D, and S2172T) were grown at The Land Institute (Salina, Kansas, USA) in 2018 and 2019 using a randomized design with 2 blocks each year. In 2018, unfavorable weather necessitated 2 planting dates (June 5 and June 8), and planting date was modeled as a block effect. In 2019, all entries were sown on May 20.

FT was recorded for each row as the number of days from planting until approximately 50% of plants in that row had flowered. After quality control for both genotypes and phenotypes, complete data were available for 179 individuals in S2039D, 81 in S2039T, 200 in S2172D, and 53 in S2172T.

### Parental analysis and run-length methodology

To characterize genomic introgression patterns in the tetraploid intermediates, we retained 13,991 SNPs segregating across 5 genotypes: the cultivated *S. bicolor* parent (K105), 2 *S. halepense* accessions (G9N and G2012), and the tetraploids T115 and T254. G9N and G2012 are the actual *S. halepense* accessions used as parents in the original backcrossing program that produced T254 and T115, respectively, and were therefore used here as the primary reference for inferring halepense-derived ancestry. The annual inbred KS105A (*S. bicolor*; USDA GRIN accession PI 612408) served as the cultivated reference parent (K105). Sites polymorphic in K105 relative to the *S. bicolor* BTx623 reference genome were excluded so that departures from the reference state would primarily reflect *S. halepense*-derived variation rather than standing polymorphism within cultivated sorghum. This filtering strategy ensured that subsequent inference of allele origin was anchored to a consistent cultivated reference background.

For each retained SNP, we defined a composite indicator of *S. halepense* ancestry, $G_{\mathrm{combined}}$. This indicator was coded as 0 if both G9N and G2012 matched the BTx623 reference allele, and 1 if at least one of the two *S. halepense* accessions carried an alternative allele. Thus, $G_{\mathrm{combined}}=1$ identified loci with evidence of halepense-derived variation relative to the cultivated reference.

Genotypes in T115 and T254 were coded as 0 (homozygous reference), 1 (heterozygous), or 2 (homozygous alternative) with respect to BTx623. Within each tetraploid genome, markers were ordered by physical position and scanned sequentially to identify consecutive loci sharing the same genotype class. Such consecutive segments were treated as “runs” of class 0, 1, or 2, representing putative contiguous chromosomal tracts of shared ancestry.

To determine whether observed runs exceeded expectations under random marker ordering, we applied the run-length framework of Kong et al. (2020). For genotype class *g*, the minimum run length $k_{g}$ considered unlikely under random segregation was defined by


$$1-\left[{\left(\frac{n_{g}}{N}\right)}^{k_{g}}(N+1-k_{g})\right]\geq 1-\alpha ,$$

where *N* denotes the total number of markers, $n_{g}$ the number of markers in class *g* genome-wide, and α=0.05. Runs exceeding this threshold were interpreted as nonrandom clusters of homozygous or heterozygous segments. Such extended tracts are consistent with introgressed chromosomal regions or regions subject to selection or transmission bias during the formation of halepense-derived tetraploids.

### SNP calling and filtering in S2039 and S2172

For joint analysis of diploid and tetraploid progeny, variants were called across all individuals under a tetraploid genotype model to facilitate comparisons based on dosage. Only biallelic SNPs with minor allele frequency (MAF) greater than 0.05 were retained.

Depth filters were applied to reduce spurious homozygous calls. Specifically, homozygous genotypes with read depth <6 were masked, as low-depth homozygous calls were considered unreliable for distinguishing true homozygotes from miscalled heterozygotes. Markers with more than 20% missing data across individuals were removed. To minimize redundancy, markers in near-perfect linkage (r>0.99) were identified, and one representative per highly correlated set was retained. After filtering, 10,102 SNPs for S2039 and 5,945 SNPs for S2172 remained, and genotypes were expressed as tetraploid dosage classes (0–4).

### Linear discriminant analysis for ploidy classification

To distinguish diploid from tetraploid individuals using genotype data, we summarized each individual by the per-individual proportions of markers called as simplex (dosage class 1) and triplex (dosage class 3) under the tetraploid calling model. These 2 quantities were used as predictors in a linear discriminant analysis (LDA), with cytologically verified diploid or tetraploid status as the response, to evaluate how well dosage profiles alone separate the 2 ploidy classes. Inspection of dosage-class distributions indicated that simplex and triplex frequencies provided the clearest separation between ploidy classes.

### Multidimensional scaling of genomic relationships

For each family, we constructed a combined genotype matrix spanning both diploid and tetraploid individuals, using dosage-based calls and removing markers with r>0.99 to reduce redundancy. For distance-based analyses, genotypes were recoded into 3 classes: 0 (homozygous reference), 1 (heterozygous; dosage 1–3), and 2 (homozygous alternative). Pairwise genomic distances between individuals *i* and *j* were then computed using a modified Hamming distance:


$$d_{ij}=\frac{1}{2m^{\prime }}\sum_{k=1}^{m}|x_{ik}-x_{\;jk}|,$$

where $m^{\prime }$ is the number of markers scored in both individuals (ie nonmissing in *i* and *j*), and $x_{ik}$ and $x_{\;jk}$ denote recoded genotypes at marker *k*. Classical multidimensional scaling (MDS) of the resulting distance matrix was performed using the cmdscale function in R (RRID:SCR_001905).

### Comparative genomic composition of diploid and tetraploid progeny

To compare genomic composition between diploid and tetraploid progeny within each family, we estimated the alternative-allele frequency (*p*) for every SNP in each population and assigned it to one of three classes: *S. bicolor*-enriched (p<0.25; g=0), mixed (0.25≤p≤0.75; g=1), or *S. halepense*-enriched (p>0.75; g=2). As above, we identified runs of consecutive markers sharing the same class and applied run-length thresholds derived from the global frequency of each class to identify unusually long segments. The distribution of markers among these 3 categories in each population is summarized in Table 1.

**Table 1.** Distribution of allele-enrichment groups in diploid (D) and tetraploid (T) S2039 and S2172 populations. Groups are defined by alternative-allele frequency *p*: *S. bicolor*-enriched (g=0, p<0.25), equal contribution (g=1, 0.25≤p≤0.75), and *S. halepense*-enriched (g=2, p>0.75). Values represent marker counts.

Group	S2039D	S2039T	S2172D	S2172T
*S. bicolor* enriched (g=0)	6,401	1,690	2,273	1,103
Equal contribution (g=1)	3,114	7,922	3,020	4,701
Alternative-allele enriched (g=2)	587	490	652	141
Total markers	10,102	10,102	5,945	5,945

### Genetic map construction

For QTL mapping in diploid families, we applied additional quality filters tailored to linkage analysis. GATK hard-filter criteria were used at the variant level:

- QD < 2.0 || FS > 60.0 || MQ < 40.0 ||
- MQRankSum < -12.5 || ReadPosRankSum < -8.0

**Fig. 3.**
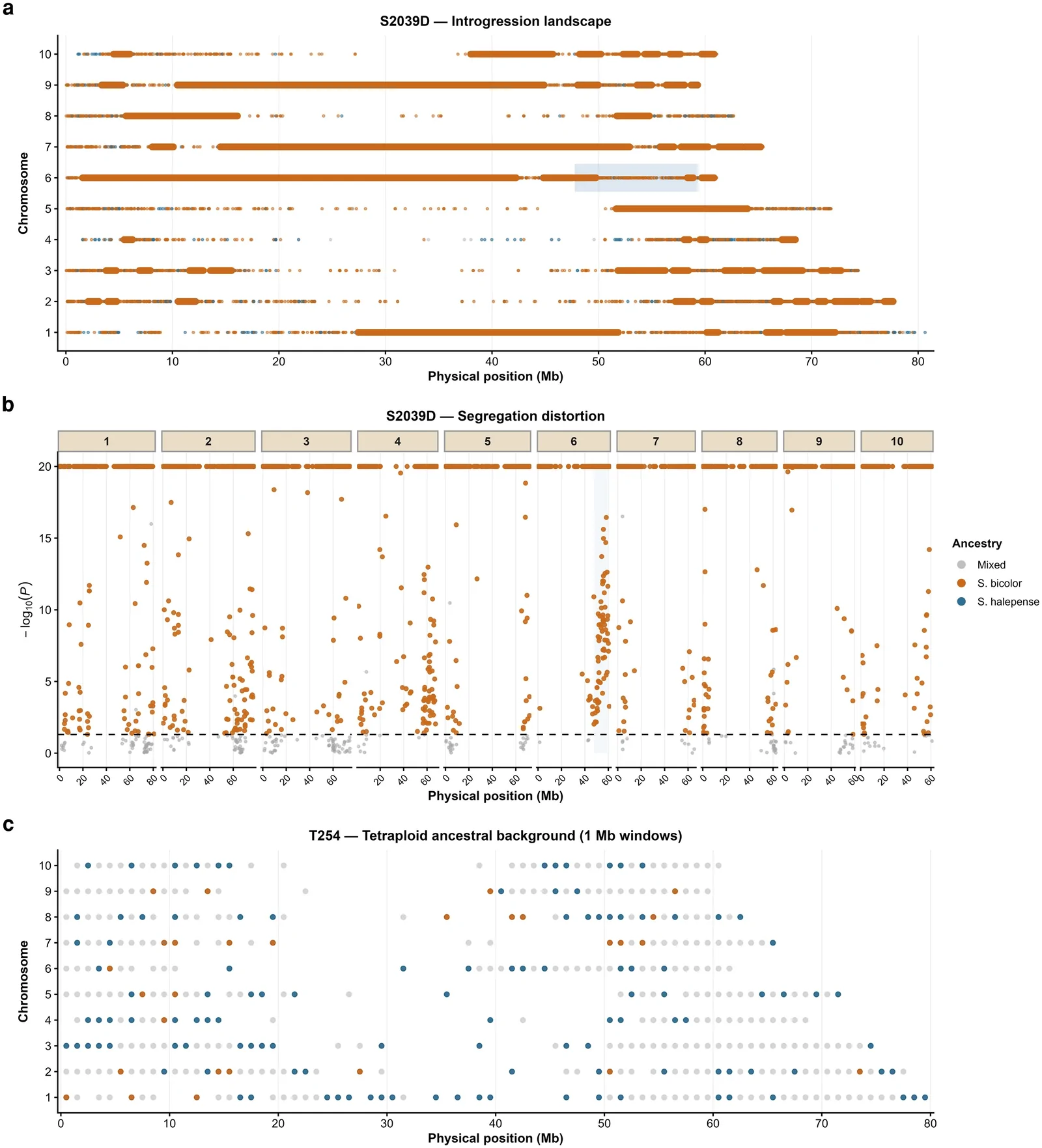
Genomic composition and segregation distortion in the S2039 family. a) Introgression landscape of S2039D across chromosomes 1–10. Points are colored by ancestry class (orange: *S. bicolor*-enriched, p<0.25; blue: *S. halepense*-enriched, p>0.75; gray: mixed); orange bars mark *S. bicolor*-enriched runs (≥27 markers). Blue shading on chromosome 6 (47.76–59.34 Mb) marks a distortion interval shared with the S2172 family (Fig. 4). b) Segregation distortion in S2039D plotted as $-\log_{10}\;(p)$ from $\chi^{2}$ tests against the expected 1:2:1 ratio; dashed line: p=0.05. Orange points indicate runs of ≥4 consecutive distorted markers; *y*-axis capped at 20. c) T254 tetraploid ancestral background summarized in 1 Mb windows (blue: p>0.40; orange: p<0.25). Overlap between *S. bicolor*-enriched intervals and distortion peaks suggests nonrandom genomic filtering during diploid extraction.

**Fig. 4.**
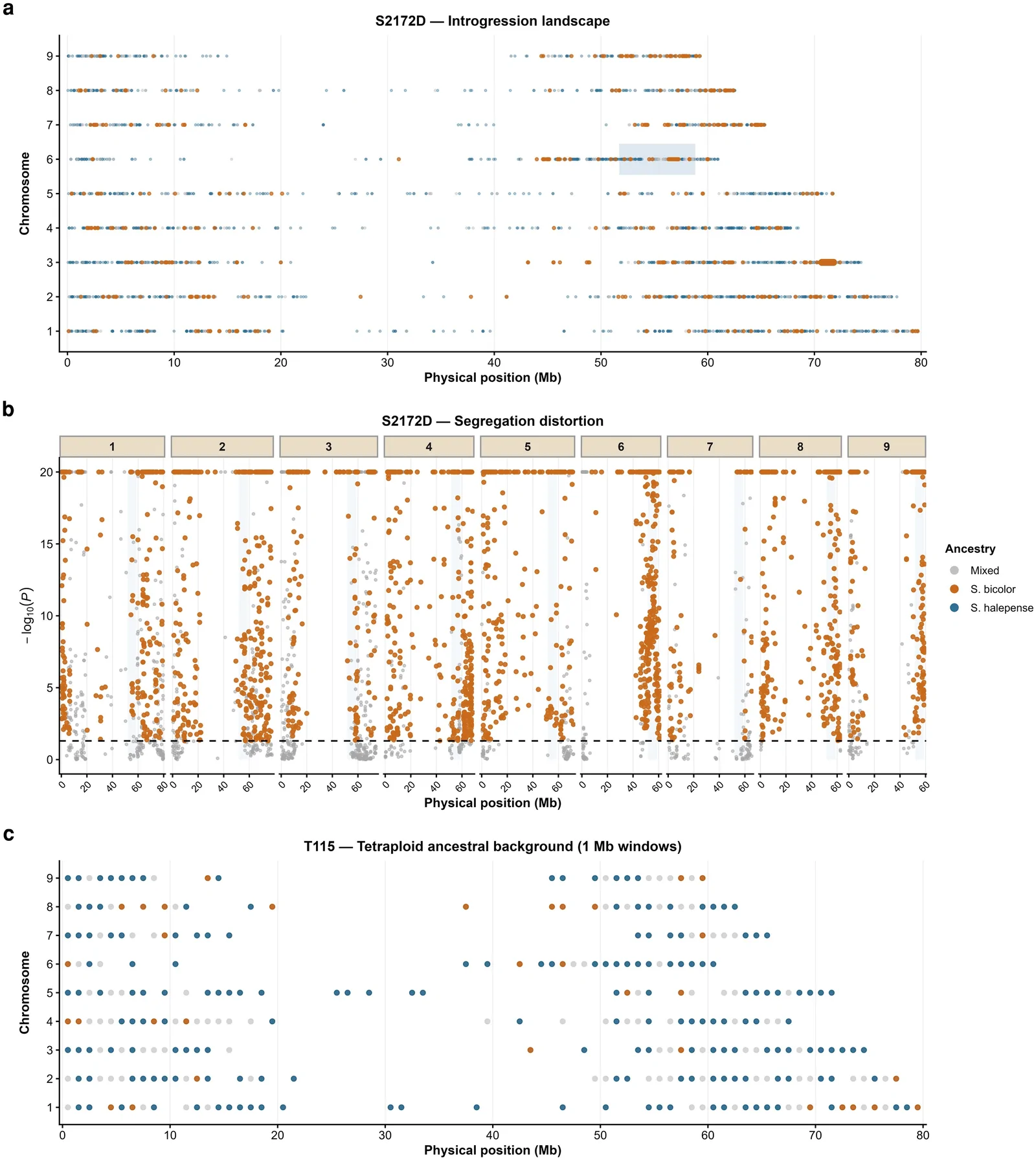
Genomic composition and segregation distortion in the S2172 family. a) Introgression landscape of S2172D across chromosomes 1–10. Points are colored by ancestry class (orange: *S. bicolor*-enriched, p<0.25; blue: *S. halepense*-enriched, p>0.75; gray: mixed); orange bars mark *S. bicolor*-enriched runs (≥13 markers). Blue shading on chromosome 6 (51.71–58.83 Mb) marks a distortion interval shared with the S2039 family (Fig. 3). b) Segregation distortion in S2172D plotted as $-\log_{10}\;(p)$ from $\chi^{2}$ tests against the expected 1:2:1 ratio; dashed line: p=0.05. Orange points indicate runs of ≥6 consecutive distorted markers; *y*-axis capped at 20. c) T115 tetraploid ancestral background summarized in 1 Mb windows (blue: p>0.40; orange: p<0.25). Overlap between *S. bicolor*-enriched intervals and distortion peaks suggests nonrandom genomic filtering during diploid extraction.

SNPs with MAF ≤0.05 or more than 50% missing data were discarded. After variant-level filtering, high-density mapping datasets comprised 5,684 SNPs for S2039D and 4,697 SNPs for S2172D. These markers were used for linkage-group assignment and initial recombination fraction estimation in onemap (Margarido et al. 2007). For final map construction and downstream R/qtl analyses (Broman et al. 2003) (RRID:SCR_001821), we retained reduced framework marker sets to minimize redundancy and missingness, yielding 504 mapped SNPs in S2039D (Fig. 3) and 610 mapped SNPs in S2172D (Fig. 4). Final inter-marker distances were refined in R/qtl.

### QTL mapping for FT

Quantitative trait loci (QTL) for FT in the diploid populations were detected by interval mapping (Lander and Botstein 1989) implemented in R/qtl. FT was analyzed separately for each year (2018 and 2019), using year-specific phenotypic means adjusted for block effects. Genome-wide significance thresholds for logarithm-of-odds (LOD) scores were determined by permutation testing (1,000 permutations), yielding an empirical threshold of LOD=3.5 for each year. QTL exceeding the permutation-based threshold in the initial genome scans were incorporated as additive covariates in subsequent models to refine effect estimates. Final multiple-QTL models were used to estimate additive effects and the proportion of phenotypic variance explained by each locus within each year.

Sample sizes in the tetraploid families were insufficient to support conventional linkage mapping. Accordingly, we conducted single-marker association analyses using 2018 FT data to identify loci showing evidence of association. These analyses are exploratory and are presented to facilitate qualitative comparison with QTL detected in the diploid populations, rather than for formal genome-wide inference.

### Statistical analysis

All analyses were performed in R (RRID:SCR_001905). For tetraploid single-marker tests, we report raw *p*-values in the manuscript. Sample sizes and FT summary statistics for all populations are provided in Supplementary Table S1.

## Results and discussion

### Parental genomic composition and similarity to *S. halepense*

Summary statistics in Table 2 indicate that both tetraploid intermediates, T254 and T115, retain substantial *S. halepense* ancestry while also carrying discrete tracts of alleles concordant with *S. bicolor*. At sites where the composite *S. halepense* indicator $G_{\mathrm{combined}}$ (derived from accessions G9N and G2012, the *S. halepense* parents used in the original backcrossing program) matches the *S. bicolor* reference ($G_{\mathrm{combined}}=0$), 16.0% of genotypes in T254 and 14.4% in T115 were classified as neither reference-like nor halepense-like. These “unknown” alleles likely reflect standing polymorphism within *S. halepense* not captured by the 2 parental accessions, de novo mutations arising during backcross development, or *S. bicolor* variants absent from the BTx623 reference genome. Because G9N and G2012 represent single accessions rather than population-level samples, some residual ancestry ambiguity is expected. Importantly, this uncertainty is concentrated primarily at noninformative ($G_{\mathrm{combined}}=0$) sites and does not alter the primary inference of introgression patterns based on informative ($G_{\mathrm{combined}}=1$) loci.

**Table 2.** Joint distribution of tetraploid parental genotypes (T254 and T115) and the composite *S. halepense* variable $G_{\mathrm{combined}}$. Counts are followed in parentheses by genome-wide proportions.

	$G_{combined}=0$	$G_{combined}=1$	Total
**(A) T254 vs. $G_{combined}$**			
T254 = 0	996 (0.072)	2,288 (0.165)	3,284 (0.237)
T254 = 1	2,217 (0.160)	8,331 (0.600)	10,548 (0.760)
T254 = 2	8 (0.001)	88 (0.006)	96 (0.007)
Total	3,221 (0.233)	10,707 (0.771)	13,928 (1.000)
**(B) T115 vs. $G_{combined}$**			
T115 = 0	1,191 (0.086)	4,006 (0.288)	5,197 (0.374)
T115 = 1	1,853 (0.133)	5,806 (0.417)	7,659 (0.550)
T115 = 2	159 (0.011)	863 (0.062)	1,022 (0.073)
Total	3,203 (0.230)	10,675 (0.767)	13,878 (1.000)

**Table 3.** Summary of genetic map parameters for diploid S2039D and S2172D populations. Length and spacing are in centimorgans (cM). AA, AB, and BB denote genotype frequencies for homozygous reference, heterozygous, and homozygous alternative classes, respectively.

Chr	Markers (*n*)	Length (cM)	Avg spacing (cM)	Max spacing (cM)	AA (%)	AB (%)	BB (%)
**(A) S2039D (*N* = 180)**							
1	60	261.2	4.4	20.4	27.16	47.61	25.22
2	63	249.5	4.0	11.9	30.44	47.52	22.04
3	64	252.8	4.0	9.5	25.16	49.58	25.26
4	55	198.7	3.7	10.5	32.34	47.73	19.93
5	48	211.4	4.5	12.5	26.87	47.71	25.42
6	42	135.4	3.3	8.4	16.79	48.50	34.71
7	43	200.9	4.8	15.7	28.82	45.78	25.40
8	36	185.6	5.3	16.7	25.82	48.46	25.72
9	51	197.0	3.9	13.4	28.02	49.50	22.48
10	42	202.9	4.9	10.9	27.90	46.45	25.65
Overall	504	2,095.6	4.28	12.99	$27.19^{a}$	47.94	24.86
**(B) S2172D (*N* = 300)**							
1	73	364.2	5.1	37.0	27.30	45.80	26.89
2	84	323.5	3.9	15.8	28.11	46.20	25.69
3	90	356.7	4.0	15.0	26.36	48.16	25.48
4	59	270.3	4.7	15.3	30.98	46.65	22.37
5	53	285.9	5.5	12.9	29.45	47.86	22.69
6	61	240.8	4.0	10.0	22.29	46.78	30.93
7	38	161.8	4.4	11.2	25.51	51.10	23.39
8	42	167.7	4.1	10.4	22.25	45.23	32.52
9	54	251.1	4.7	11.8	28.90	46.38	24.72
10	56	249.0	4.5	22.6	30.46	44.97	24.57
Overall	610	2,671.1	4.49	16.20	27.28	46.83	25.89

^a^Percentages for the “Overall” row are weighted averages from raw genotype counts.

For sites where $G_{\mathrm{combined}}$ carries at least one alternative allele ($G_{\mathrm{combined}}=1$), the 2 tetraploids differ in the relative contributions of reference versus halepense-like haplotypes. At these loci, 16.5% (T254) and 28.8% (T115) of alleles match the *S. bicolor* reference, whereas 60.6% (T254) and 47.9% (T115) align with halepense-derived haplotypes from G9N or G2012 (Table 2). Overall, both intermediates are more similar to *S. halepense* than to *S. bicolor*, but they contain sizeable and evidently nonrandom blocks of *S. bicolor* introgression.

**Fig. 5.**
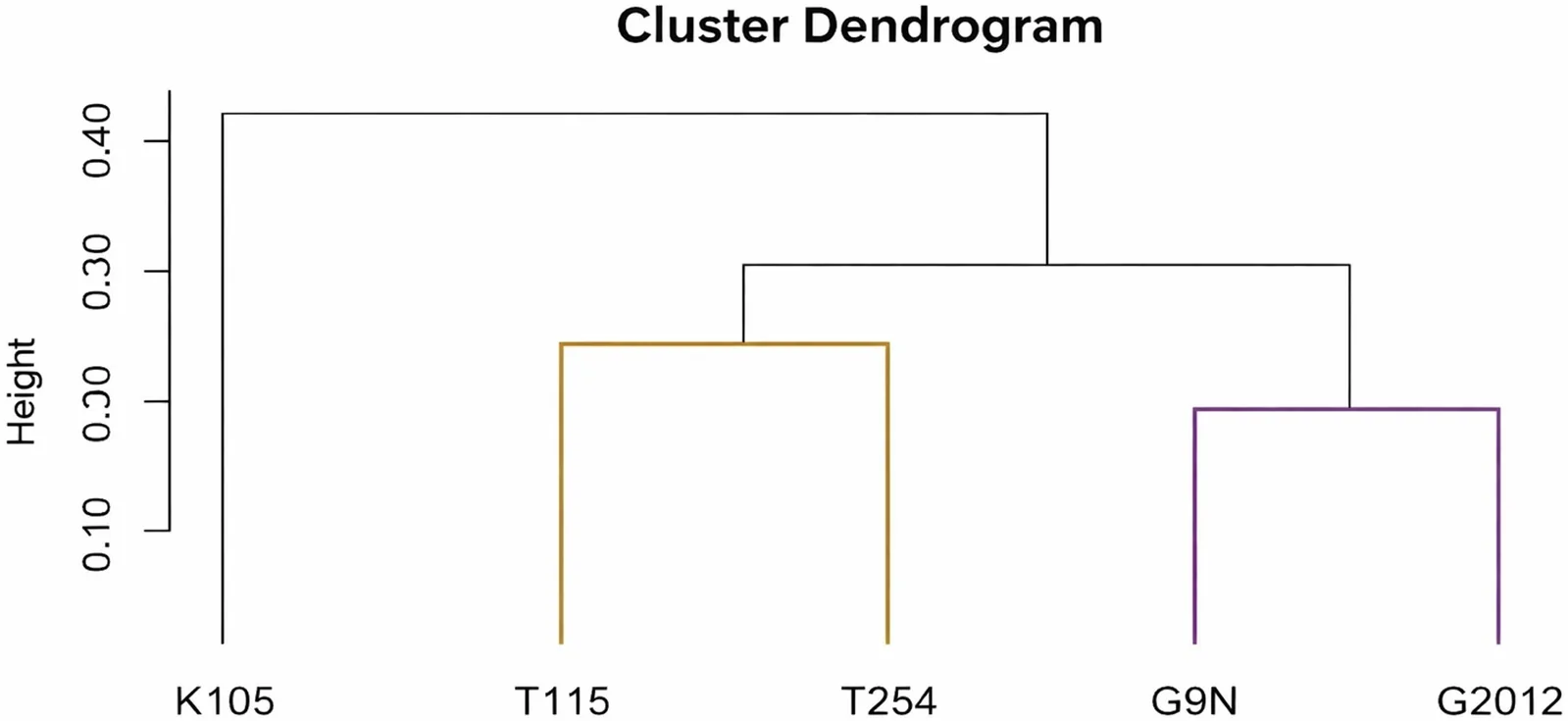
Hierarchical clustering of parental genotypes used to generate the S2039 and S2172 families. K105 represents the cultivated *Sorghum bicolor* line, whereas G9N and G2012 represent *S. halepense* accessions. T115 is the tetraploid parent of the S2172 populations, and T254 is the tetraploid parent of the S2039 populations. Clustering based on genome-wide SNP data indicates that both tetraploid intermediates group more closely with *S. halepense* than with cultivated *S. bicolor*.

Clustering of parental genotypes based on genome-wide SNP data (Fig. 5) is consistent with these allele-frequency patterns. T115 and T254 group more tightly with the halepense accessions than with cultivated *S. bicolor* K105, reinforcing the view that recurrent introgression has not erased the fundamentally halepense-like character of the tetraploid genomes.

Run-length analysis further resolves how *S. bicolor*-like tracts are distributed along chromosomes in the tetraploid parents. In T115, 8 regions exceed the threshold for unusually long runs of homozygous *S. bicolor*-like alleles (minimum run length k=13, p<0.05; Supplementary Fig. S1), whereas T254 carries 2 such regions (Supplementary Fig. S2). Several of these tracts coincide with a region on chromosome 5 that includes the rDNA locus previously reported to be depleted for *S. halepense* alleles (eg Kong et al. 2020). Recurrence of this pattern across independent interspecific backgrounds suggests that a particular *S. bicolor* haplotype in the rDNA region has been consistently favored during the development of halepense-derived tetraploids.

### Comparative genomic composition of diploid and tetraploid progeny

**Fig. 6.**
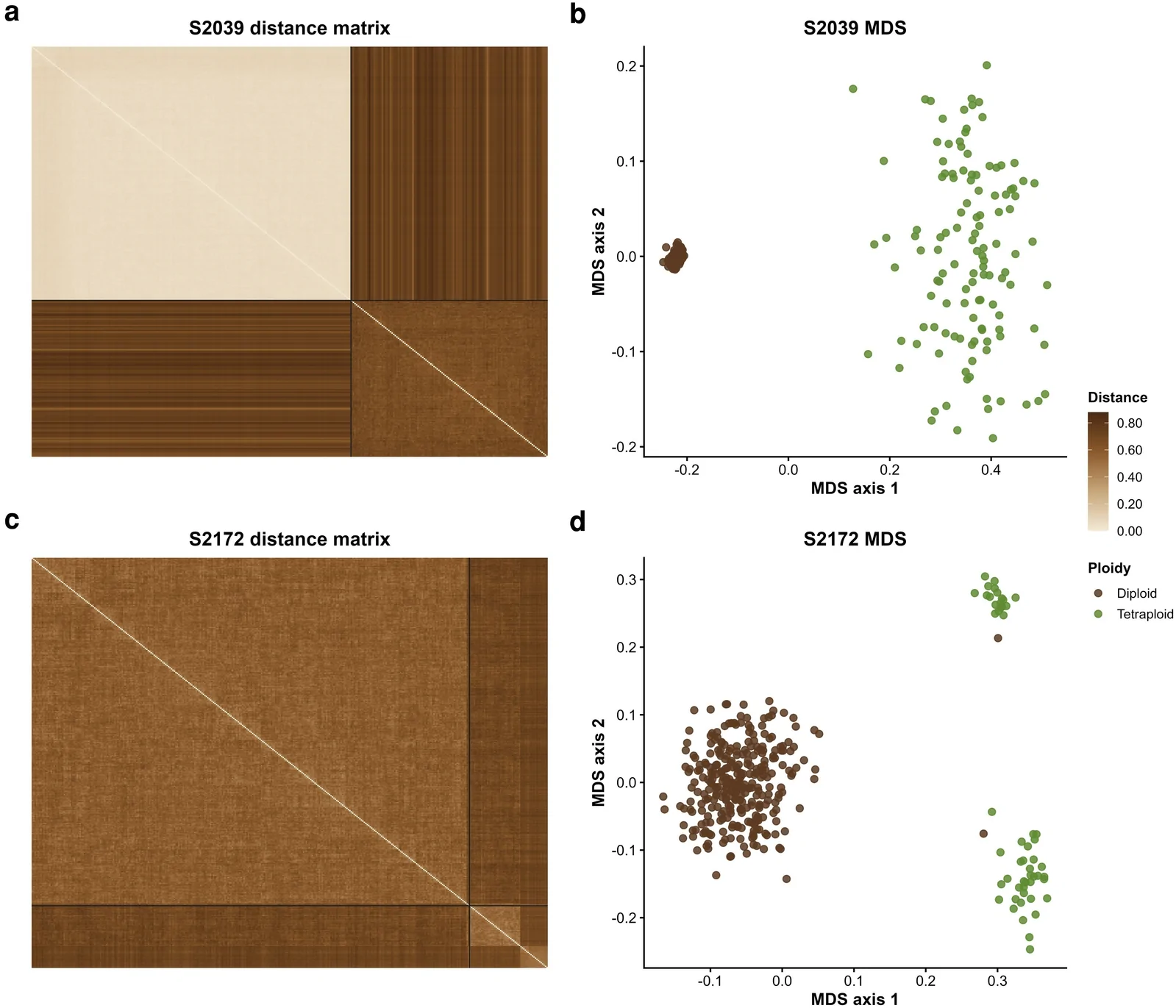
Genomic distance structure and multidimensional scaling (MDS) analyses of diploid and tetraploid progeny in the S2039 and S2172 populations. a) and c) Pairwise modified Hamming distance matrices for S2039 and S2172, respectively. Individuals are ordered by ploidy class with hierarchical clustering applied within each class; warmer colors indicate greater genomic dissimilarity, and the horizontal and vertical lines demarcate the diploid–tetraploid boundary. Both matrices share a common distance color scale (0.00–0.80). b) and d) First 2 dimensions of classical MDS derived from the corresponding distance matrices, with individuals colored by ploidy class (brown: diploid; green: tetraploid). In both populations, diploid and tetraploid individuals formed clearly separated genomic clusters, supporting strong ploidy-based differentiation and providing no evidence of widespread sample misclassification. One individual in S2172T (panel d) was positioned intermediately between the main diploid and tetraploid clusters, suggesting an individual warranting further inspection.

To further characterize relationships among individuals and ploidy classes, we computed modified Hamming distances from recoded genotypes and visualized the resulting distance matrices using MDS. In both S2039 and S2172, diploid and tetraploid individuals separate cleanly along the first MDS axis (Fig. 6). This separation reinforces the conclusion that the 2 ploidy classes are genetically distinct in each family and that sample mislabeling or seed contamination is unlikely to explain the observed structure.

Shared SNPs between diploid and tetraploid subsets enabled direct comparison of genomic composition via run-length analysis. In S2039D, 5 genomic intervals (on chromosomes 1, 3 [2 regions], 5, and 6) exhibit runs of *S. bicolor*-fixed markers that exceed the significance threshold (p<0.05, k=27), while the same regions remain polymorphic in S2039T (Fig. 3). These *S. bicolor*-fixed segments coincide with large map gaps, consistent with a lack of informative polymorphism in the diploids. The largest gap, on chromosome 1, spans 9.7 cM (approximately 4.6 Mb), and another on chromosome 6 spans 7.6 cM (about 4.7 Mb).

In S2172D, 8 regions are fixed for *S. bicolor* alleles (chromosomes 1 [3 regions], 3, 6, 7 [2 regions], and 10; p<0.05, k=13), whereas the corresponding regions in S2172T remain segregating. Several of these segments align with large map gaps, including a region on chromosome 1 from 13.1 to 14.5 Mb that corresponds to a 37 cM gap, and a region on chromosome 4 from 53.7 to 56.6 Mb that coincides with a 15 cM gap. One fixed region on chromosome 3 lies within pericentromeric heterochromatin, suggesting that alternative alleles are retained in tetraploids but not in the derived diploids (Fig. 4).

Across both families, we identified 13 genomic intervals that segregate in tetraploids but are fixed for *S. bicolor* alleles in diploids, a pattern unlikely to arise under neutral segregation given the overall allele frequencies (p<0.05 genome-wide). These biases suggest that some *S. halepense*-derived alleles tolerated in a polyploid genomic background may become deleterious or strongly disfavored when returned to diploidy. Possible mechanisms include dosage sensitivity, subfunctionalization dependent on homoeologous gene copies, or epistatic interactions that require a polyploid genomic context. Another possibility is that some loci carry partially recessive or loss-of-function variants that are buffered by homoeologous gene copies in the tetraploid state but become exposed and selectively disfavored following diploidization. Distinguishing among these mechanisms will require targeted functional analyses of candidate loci within the affected genomic intervals.

### Marker distributions and ploidy classification

**Fig. 7.**
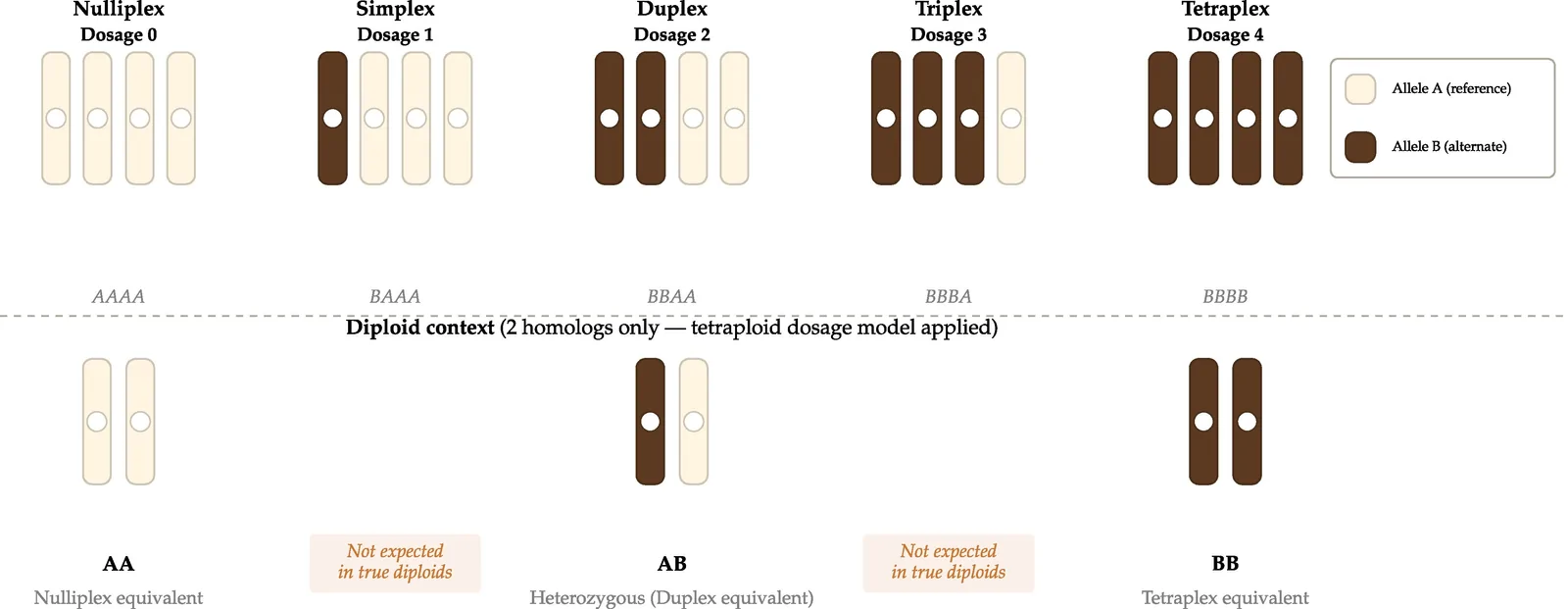
Conceptual representation of SNP dosage classes under the tetraploid genotype-calling framework used in this study. Dosage classes range from nulliplex (dosage 0) to tetraplex (dosage 4), representing the number of alternative alleles across four homologous chromosomes. Equivalent diploid genotype states are shown below. Under diploid segregation, only nulliplex, duplex, and tetraplex states are expected, whereas simplex and triplex states are diagnostic of tetraploid dosage structure.

Across the joint diploid–tetraploid genotype calls, 10,102 markers were retained in S2039 and 5,945 in S2172. The smaller marker set in S2172 likely reflects lower effective polymorphism in that family, exacerbated by the smaller tetraploid sample. Because variants were called under a tetraploid model for all individuals, we asked whether ploidy could be recovered solely from dosage profiles. Under this calling framework, 5 dosage classes are possible (Fig. 7): nulliplex (0), simplex (1), duplex (2), triplex (3), and tetraplex (4), representing 0 through 4 copies of the alternative allele across four homologs. In the absence of genotyping or dosage-calling error, diploid genotypes called as tetraploids should be confined to three of these classes—nulliplex (0; homozygous reference), duplex (2; effective heterozygote), and tetraplex (4; homozygous alternative)—with expected frequencies of 0.25, 0.50, and 0.25, respectively. Simplex and triplex calls are not expected in true diploids and therefore provide a direct signal of tetraploid dosage structure. In the observed data, simplex and triplex calls are infrequent but not absent (3.1% and 2.7% in S2039D; 4.6% and 4.0% in S2172D), consistent with occasional misclassification of heterozygotes and providing a rough bound on genotype-calling error.

**Fig. 8.**
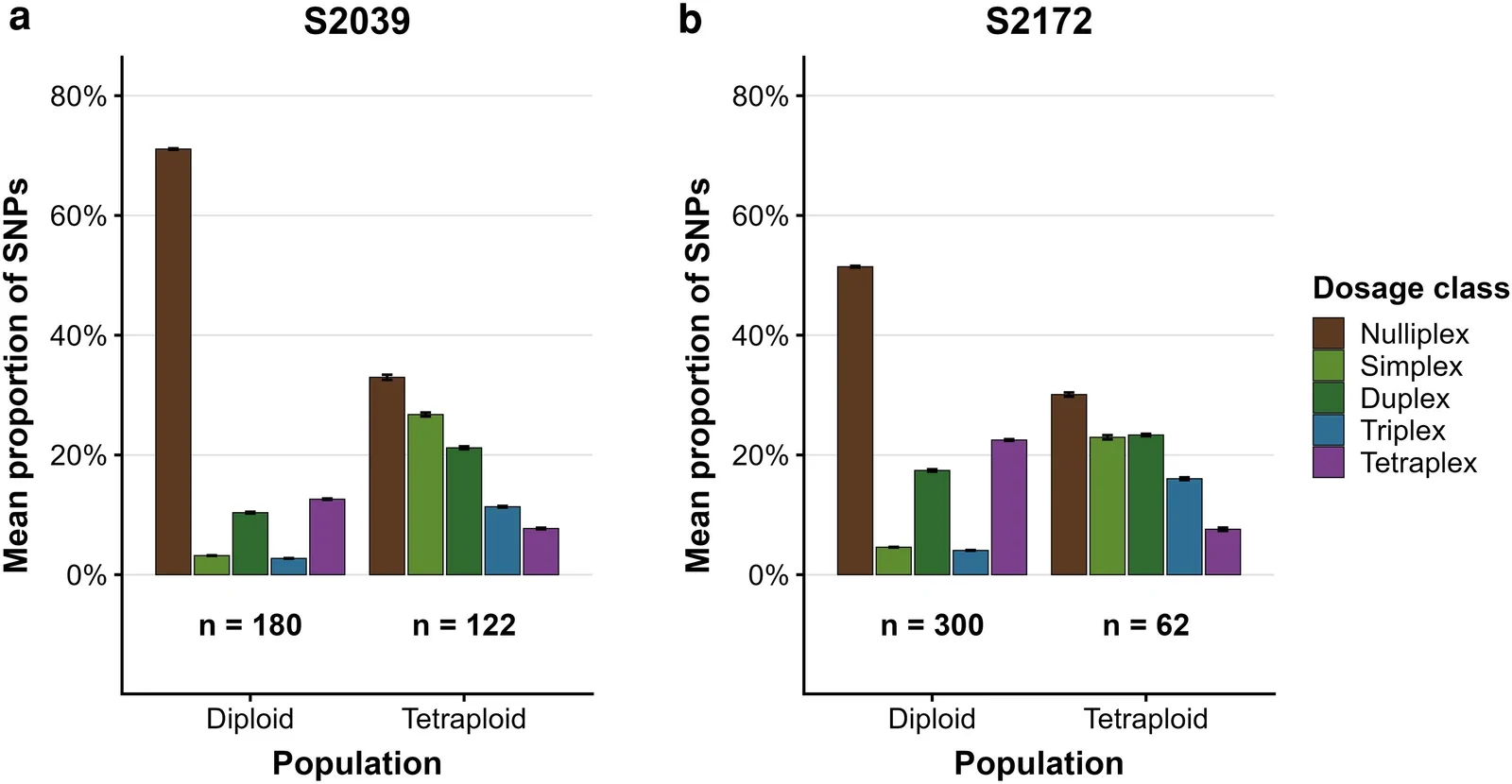
SNP dosage-class distributions in diploid and tetraploid progeny derived from the S2039 and S2172 populations. a) Comparison of S2039D and S2039T. b) Comparison of S2172D and S2172T. Bars represent the mean proportion of SNPs assigned to each dosage class (nulliplex, simplex, duplex, triplex, and tetraplex) under a tetraploid dosage-calling framework, with error bars representing standard errors across individuals. Values beneath the D and T groups indicate the number of individuals (*n*) within each ploidy class. Relative to tetraploid populations, diploid populations exhibit substantially higher proportions of nulliplex calls and markedly reduced frequencies of intermediate dosage classes, particularly simplex and triplex genotypes. These dosage-profile differences provided the basis for ploidy discrimination in subsequent analyses.

Nevertheless, the diploid populations show markedly lower proportions of simplex and triplex calls than their paired tetraploid populations (Fig. 8). Motivated by this pattern, we summarized each individual by the per-individual proportions of markers called as simplex and triplex and used these 2 quantities as predictors in a LDA. Inspection of dosage-class distributions (Fig. 8) confirmed that simplex and triplex frequencies provide the clearest separation between ploidy classes, consistent with the expectation from Fig. 7.

The resulting discriminant function was


$$\begin{array}{rl} \mathrm{Score}\;= & -9.1856+41.6116\times \;\text{prop( Simplex) }+36.8495\\ & \times \;\text{prop( Triplex) } \end{array}.$$

Discriminant scores ranged from −5.20 to −0.30 among diploids and from 4.04 to 12.07 among tetraploids, yielding complete separation of the 2 ploidy classes in this dataset. These results show that ploidy can be inferred with high accuracy from dosage-based summaries once GBS or similar SNP data are available, providing a practical complement to flow cytometry or chromosome counting in related interspecific sorghum populations.

### FT variation and QTLs across ploidy levels

Population means and standard deviations for FT are given in Table S1. In the S2039 families, tetraploids (S2039T) flower considerably later than diploids (S2039D), by 15.80 d in 2018 and 11.02 d in 2019, consistent with reports that polyploids often show delayed reproduction relative to diploids (eg Petit et al. 1997). In contrast, for S2172 the tetraploid population (S2172T) flowers earlier than the diploid S2172D by 6.36 d (2018) and 4.55 d (2019), illustrating that the direction of ploidy effects on FT is highly dependent on the allelic composition of the pedigree.

**Fig. 9.**
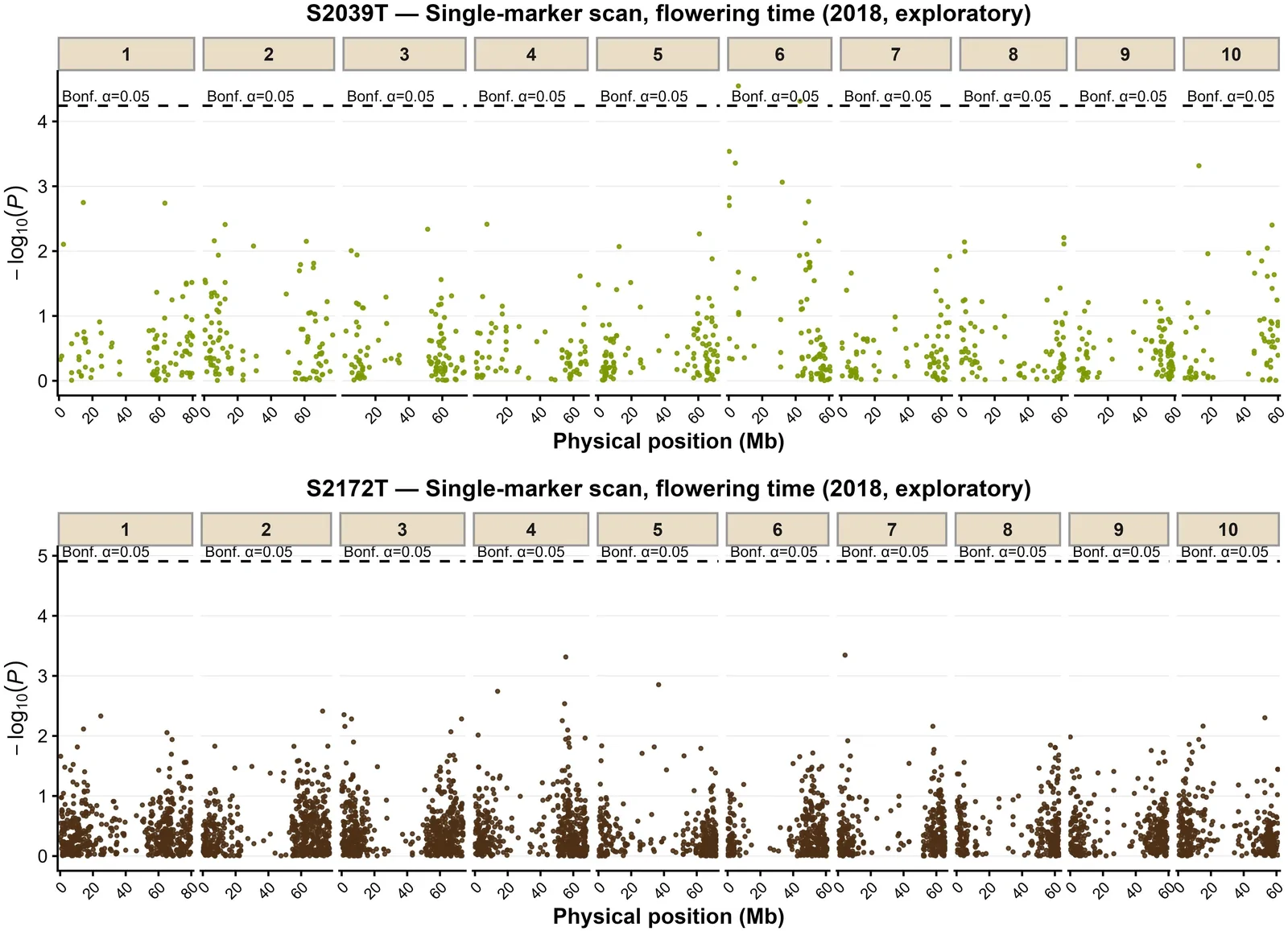
Single-marker association scans for FT (2018) in tetraploid interspecific families. Each point represents a SNP marker plotted as $-\log_{10}\;(p)$ from a linear model testing genotype-class effects on FT, ordered by physical position and faceted by chromosome (1–10). a) S2039T (KS102A × T254>123). b) S2172T (KS105A × T115>B29A). Phenotypes are genotype-level estimated marginal means (EMMs) for 2018 FT, derived from linear models block-adjusted where replication structure permitted. The dashed horizontal line indicates the Bonferroni-corrected significance threshold (α=0.05, per-panel). No marker exceeded the genome-wide threshold in either population, consistent with limited statistical power in these family sizes; visual peaks on chromosomes 5 and 6 of S2039T may motivate future investigation.

In S2039D, 3 QTLs for FT (qFL6.1, qFL9.1, and qFL10.1) exceed the permutation-based threshold, with qFL9.1 and qFL10.1 detected in both years (Table 4). In S2039T, single-marker scans identify 2 prominent association peaks on chromosome 6 (near 1.0 Mb and 47.1 Mb) but do not show strong signals near the diploid QTLs on chromosomes 9 or 10 (Fig. 9). The S2039D QTL qFL6.1 maps roughly 9 Mb away from the dominant S2039T signal on chromosome 6, leaving open whether these signals reflect distinct loci or a shared causal region that is poorly resolved in one or both populations. Overall, the results suggest that FT in S2039 is governed by different combinations of loci in diploid and tetraploid backgrounds.

**Table 4.** Flowering time (FT) QTL detected in S2039D and S2172D populations across 2 years. LOD, location (Loc; cM), physical position (Pos; Mb), variance explained (Var; %), additive effect (*A*)^*a*^, dominance effect (*D*)^*b*^, and physical support interval (Start–End; Mb) are shown together with candidate genes or previously reported QTL and references.

Pop	Year	QTL	LOD	Loc (cM)	Pos (Mb)	Var (%)	*A*	*D*	Start (Mb)	End (Mb)	Candidate gene/QTL	Reference
S2039D	2018	qFL9.1	9.4	90.0	50.73	18.48	−1.74	−0.49	46.79	52.49	qFL9.1 (*S. propinquum*)	Kong et al. (2013)
S2039D	2018	qFL10.1	7.0	85.2	12.12	13.38	1.46	−0.26	7.41	47.25	*CO*	Yang et al. (2014)
S2039D	2019	qFL6.1	3.9	129.0	58.79	6.21	−1.06	0.36	57.76	59.34	qFL6.1 (IS3620C)	Kong et al. (2018)
S2039D	2019	qFL9.1	10.6	105.0	52.49	18.36	−1.82	−1.10	50.45	52.79	qFL9.1 (*S. propinquum*)	Kong et al. (2013)
S2039D	2019	qFL10.1	7.7	83.0	9.67	12.77	1.67	0.19	6.82	49.46	*CO*	Yang et al. (2014)
S2172D	2018	qFL1.1	7.5	160.3	55.05	6.60	−1.19	−1.11	53.49	55.83	qFL1.1 (IS3620C)	Kong et al. (2018)
S2172D	2018	qFL4.1	5.4	13.0	2.64	4.63	1.23	0.04	0.42	4.23	qFL4.1 (*S. propinquum*)	Kong et al. (2013)
S2172D	2018	qFL4.2	5.6	227.5	62.99	4.77	−1.29	0.92	62.42	63.23	qFL.4D.H6.1 (*S. halepense*)	Kong et al. (2021)
S2172D	2018	qFL6.1	8.6	111.2	50.62	7.61	−1.67	0.13	50.18	50.94	–	–
S2172D	2018	qFL9.2	24.2	251.0	59.19	26.09	2.86	0.35	58.76	59.19	*Sobic.009G257300*	Li et al. (2018)
S2172D	2018	qFL10.1	12.1	68.0	6.85	11.25	1.92	−0.80	5.79	7.62	Maturity QTL	Ritter et al. (2008)
S2172D	2019	qFL4.1	4.3	39.3	3.32	5.98	1.33	0.05	2.68	5.95	qFL4.1 (*S. propinquum*)	Kong et al. (2013)
S2172D	2019	qFL4.2	4.0	197.5	61.96	5.56	−1.45	−0.22	59.50	64.34	qFL.4D.H6.1 (*S. halepense*)	Kong et al. (2021)
S2172D	2019	qFL6.1	6.1	117.6	50.94	8.65	−1.66	−0.43	50.62	51.71	–	–
S2172D	2019	qFL9.2	7.4	249.0	59.19	10.53	1.84	0.12	57.81	59.19	*Sobic.009G257300*	Li et al. (2018)
S2172D	2019	qFL10.1	5.5	53.1	5.16	7.75	1.45	−1.40	4.61	8.53	QDTF_S7_10.1	Wang et al. (2014)

^*a*^ Additive effect (*A*) is defined as half the difference between mean homozygotes.

^*b*^ Dominance effect (*D*) is defined as the deviation of the heterozygote from the mid-point of homozygotes.

The S2172D population harbors 6 FT QTLs, 5 of which are detected in both years (Table 4). The largest-effect locus, qFL9.2, explains 26.09% of the phenotypic variance in 2018 and 10.53% in 2019, and maps approximately 7 Mb from qFL9.1 in S2039D. Interestingly, the chromosome 9 QTLs show opposite additive effects across families: in S2039D, the *S. halepense* allele is associated with earlier flowering, whereas in S2172D it is associated with delayed flowering. This sign reversal points to strong background dependence in the phenotypic expression of alleles in this region.

No FT QTLs in S2172T exceeded the exploratory significance threshold, which is not unexpected given the relatively small tetraploid sample size (n=53). Notably, loci corresponding to the major diploid QTLs qFL9.2 and qFL10.1 did not show conspicuous association peaks in the tetraploid data. Together with the S2039 results, these patterns are consistent with the possibility that FT architecture differs between diploid and tetraploid backgrounds, even when families share substantial portions of their parental genomes. However, the relatively small tetraploid sample sizes (S2039T, n=81; S2172T, n=53) limit the strength of this inference.

Several of the FT QTLs identified here co-localize with regions highlighted in the Sorghum QTL Atlas (Mace et al. 2019). In particular, QTLs on chromosomes 9 and 10 map near candidate genes including *ELF3*- and *CONSTANS*-like loci, which have been implicated in sorghum photoperiod sensitivity and floral initiation (Yang et al. 2014; Li et al. 2018). These positional correspondences strengthen confidence in the QTL signals and highlight the utility of diploid extractions for connecting classical FT loci to variation present in polyploid-derived germplasm.

### Implications for diploidization, reproductive isolation, and breeding

Diploid families recently derived from polyploid progenitors are uncommon, and the S2039D and S2172D populations provide a rare window into transmission genetics at the onset of post-polyploid diploidization. The diploid progenitors of *S. halepense* are estimated to have diverged 1–2 million years ago (Paterson et al. 1995), placing the origin of the polyploid squarely within the timescale over which biased fractionation, structural change, and gene loss are expected following whole-genome duplication (Lynch and Conery 2000; Mandáková and Lysak 2018). Comparisons between diploid and tetraploid populations derived from the same tetraploid parents suggest that some alleles captured in the polyploid are poorly tolerated or strongly selected against when returned to diploidy.

The shared enrichment of *S. halepense* alleles and segregation distortion on chromosome 6, together with multiple *S. bicolor*-fixed regions that segregate in tetraploids but not in diploids, points to a continuum of evolutionary outcomes. At one end are alleles that appear compatible across ploidy levels and introgress readily; at the other are alleles that may be dosage-sensitive, subfunctionalized, or embedded in polyploid-specific regulatory networks that break down in a diploid context. The mixed pattern of retention and loss we observe is consistent with a model in which polyploid *S. halepense* has accumulated novel combinations of alleles and regulatory interactions, some of which are effectively locked into a polyploid genome organization.

These findings have both conceptual and applied consequences. From an ecological and biosafety perspective, *S. halepense* is often cited as a classic case of crop–weed gene flow and a potential route for transgene escape. Genomic features that reduce the fitness of particular *S. bicolor* × *S. halepense* genotypes, or that restrict the viability of certain allele combinations in diploids, may mitigate such risks, whereas polyploid-derived alleles that confer strong fitness advantages (eg rhizomatousness or reproductive barriers) could be harnessed in strategic containment schemes (Gressel 2015).

At the same time, the diploid extractions studied here offer a practical avenue to exploit the evolutionary novelty of *S. halepense* for crop improvement. Traits such as perenniality, altered flowering behavior, yield components, and possibly interactions with soil biota can now be studied in a diploid framework accessible to standard genetic dissection (Glover et al. 2010; Wang et al. 2015; Mace et al. 2019). However, direct integration of these lines into elite sorghum breeding programs would be premature. The genome structures of diploid progeny derived from polyploid intermediates cannot be expected to resemble cultivated *S. bicolor*, which is likely to impair chromosomal pairing and recombination during meiosis—the processes that plant breeders depend on to break linkage disequilibrium between desirable and undesirable alleles. Furthermore, the substantial differences in FT architecture documented here between diploid and tetraploid derivatives suggest that other key physiological pathways may be similarly reconfigured following ploidy change. We therefore suggest that the diploid extractions characterized here are best positioned as early-stage trait donors, providing polyploid-derived alleles in a diploid context suitable for introgression into diverse breeding backgrounds. Realizing this potential will require systematic evaluation of cross-fertility with lines from both male and female pools of elite sorghum germplasm, assessment of meiotic stability across generations, and targeted phenotypic evaluation of traits of interest (Glover et al. 2010). The ability to distinguish ploidy reliably from SNP data, combined with clonable diploid lines that capture segments of the *halepense* genome, establishes a platform for systematically testing which polyploid-derived alleles are beneficial, neutral, or deleterious when introduced into modern sorghum backgrounds (Mace et al. 2019).

## Supplementary Material

jkag165_Supplementary_Data

## Data Availability

Raw genotyping-by-sequencing (GBS) FASTQ files generated for the four study populations (S2039D, S2039T, S2172D, and S2172T) have been deposited in the NCBI Sequence Read Archive (SRA) under BioProject accession **PRJNA1412545**. The deposited records include raw sequencing reads and associated sequencing metadata. Additional supporting information associated with this study is provided in Supplementary Figs. S1, S2, and Table S1. All scripts used for linkage mapping, segregation distortion analyses, multidimensional scaling analyses, single-marker association analyses, and figure generation are publicly available through the GitHub repository: https://github.com/Pheonah/diploid-derivatives-polyploid-sorghum All data and code necessary to reproduce the analyses and results presented in this study are publicly available through the NCBI SRA repository, the supplementary materials associated with this article, and the associated GitHub repository. Supplemental material available at G3 online.
