# Supplementary material for: Diploids derived from polyploids: genetic characteristics of four novel interspecific Sorghum populations
Source: G3 (Bethesda). 2026 Jul 7;16(9):jkag165. doi: 10.1093/g3journal/jkag165 (PMC13535381; doi:10.1093/g3journal/jkag165)
Supplement: Supplementary file 1 [file jkag165_supplementary_data.pdf]

Supplementary Figure S1

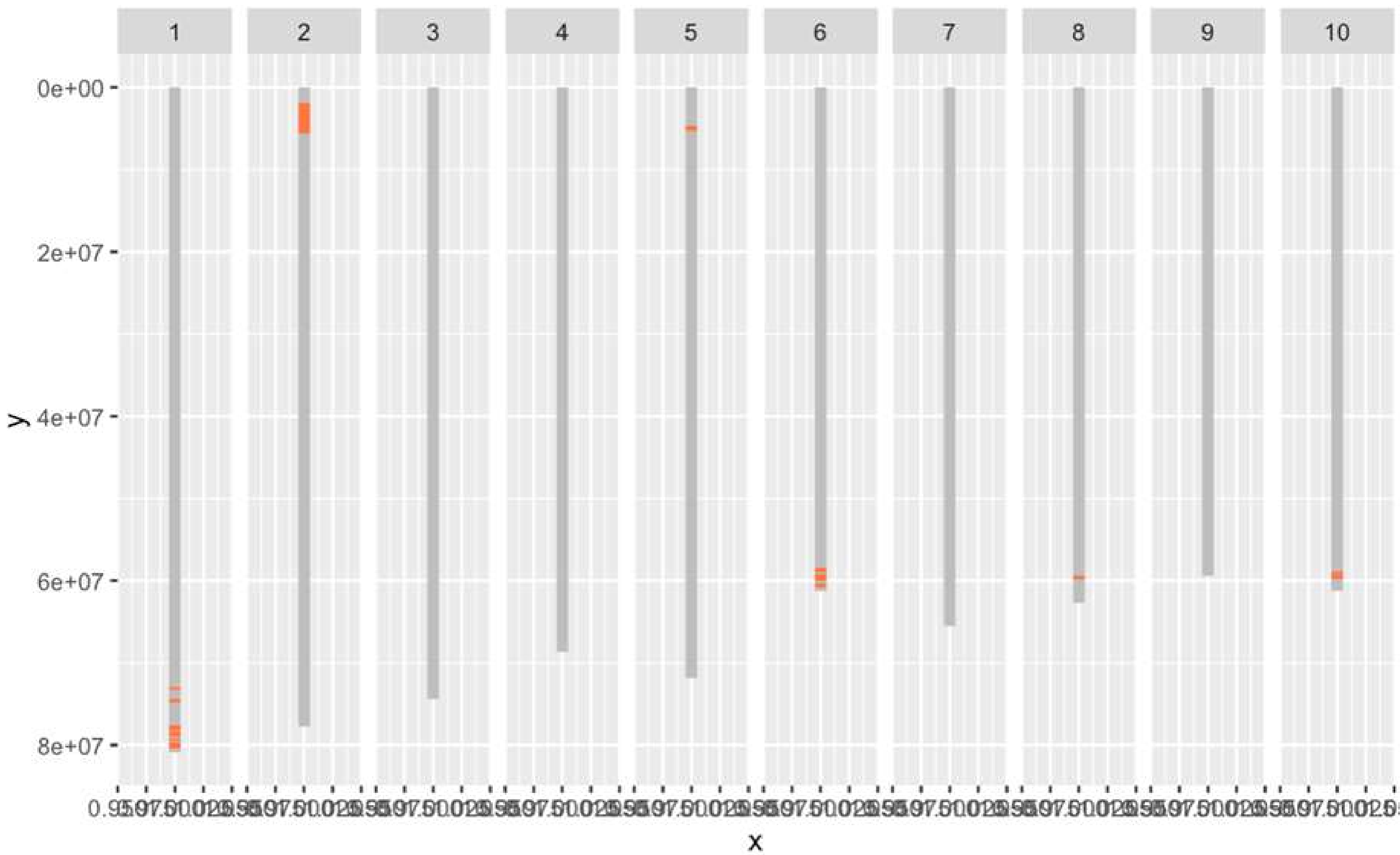

Figure S1 Genomic intervals in tetraploid parent T115 containing consecutive *S. bicolor* (SB) alleles that exceeded the run-length significance threshold ($k = 13$, $p < 0.05$). Chromosomes are shown as separate panels (1–10). The x-axis represents physical position (bp) and the y-axis corresponds to chromosome-scale genomic coordinates.

Supplementary Figure S2

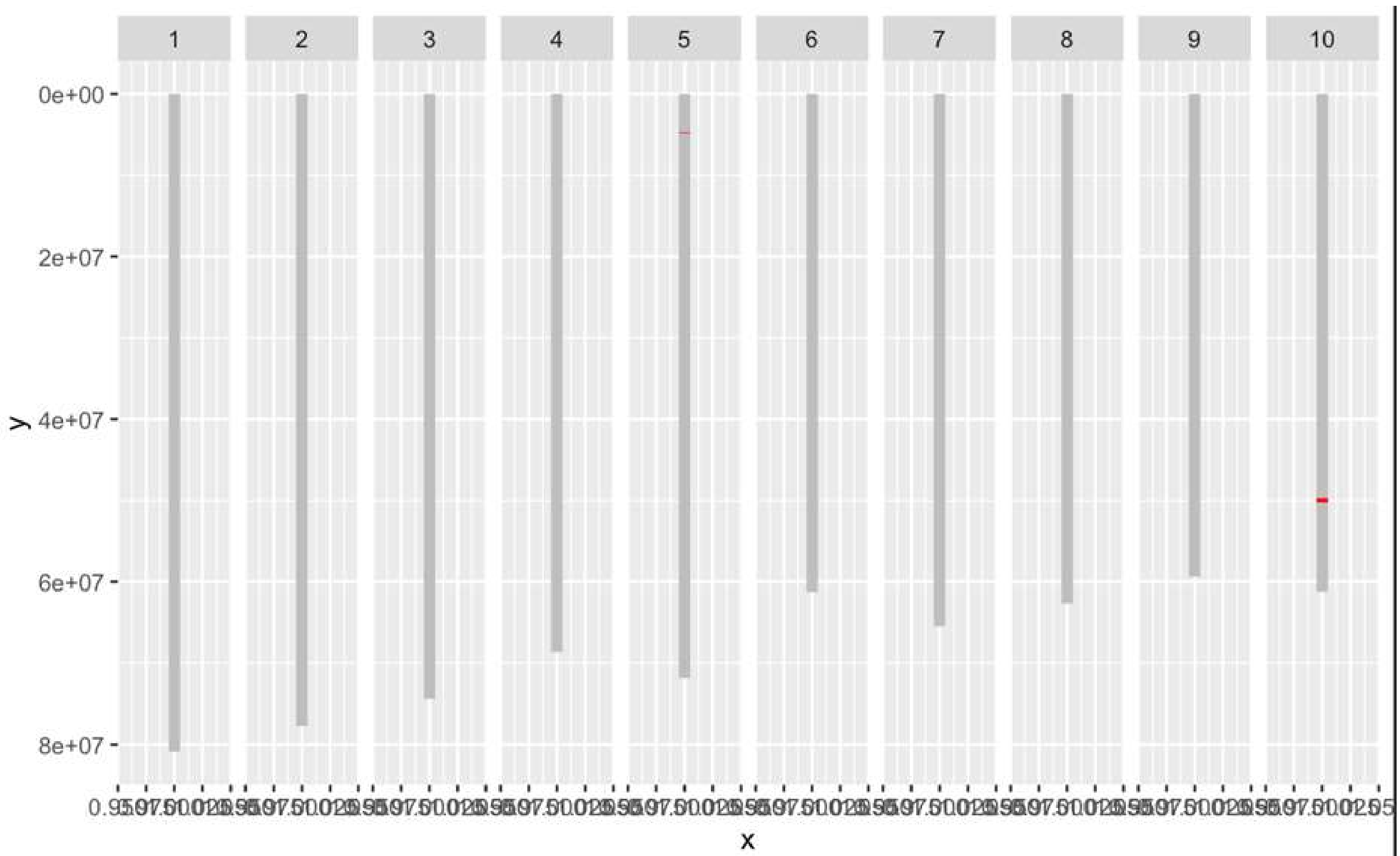

Figure S2 Genomic intervals in tetraploid parent T254 containing consecutive *S. bicolor* (SB) alleles that exceeded the run-length significance threshold ($k = 13$, $p < 0.05$). Chromosomes are shown as separate panels (1–10). The x-axis represents physical position (bp) and the y-axis corresponds to chromosome-scale genomic coordinates.

Supplementary Table S1

Supplementary Materials

Table S1. Flowering time (FT; days after planting) summary statistics for diploid and tetraploid populations across years and blocks. In 2019, a single planting date was used and block was not applicable (NA).

Year	Population	Block	N	Mean FT (days)	SD
2018	S2039D	1	164	63.80	3.27
2018	S2039D	2	159	65.52	3.21
2018	S2039T	1	40	76.68	11.61
2018	S2039T	2	41	84.12	11.52
2019	S2039D	NA	358	66.51	5.52
2019	S2039T	NA	50	77.53	9.56
2018	S2172D	1	192	68.26	4.40
2018	S2172D	2	200	70.77	4.73
2018	S2172T	1	44	60.26	9.59
2018	S2172T	2	45	65.98	7.15
2019	S2172D	NA	399	69.55	6.41
2019	S2172T	NA	16	65.00	10.35
